# Differential pH Dynamics in Synaptic Vesicles From Intact Glutamatergic and GABAergic Synapses

**DOI:** 10.3389/fnsyn.2018.00044

**Published:** 2018-12-03

**Authors:** Melissa A. Herman, Thorsten Trimbuch, Christian Rosenmund

**Affiliations:** Institute of Neurophysiology, NeuroCure Cluster of Excellence, Charité-Universitätsmedizin, Berlin, Germany

**Keywords:** vesicular transporter, proton gradient, VGLUT, VGAT, synaptic vesicle

## Abstract

Synaptic transmission requires the presynaptic release of neurotransmitter from synaptic vesicles (SVs) onto the postsynaptic neuron. Vesicular neurotransmitter transporter proteins, which use a V-ATPase-generated proton gradient, play a crucial role in packaging neurotransmitter into SVs. Recent work has revealed different proton dynamics in SVs expressing the vesicular glutamate transporter (VGLUT) or the vesicular GABA transporter (VGAT) proteins. At the whole synapse level, this results in different steady-state pH and different reacidification dynamics during SV recycling (Egashira et al., 2016). In isolated SVs, the presence of VGAT causes a higher steady state pH, which is correlated with a faster proton efflux rate (Farsi et al., 2016). To address whether proton efflux from GABAergic and glutamatergic SVs in intact synapses differs, we applied a glutamatergic- or GABAergic neuron-specific expression strategy (Chang et al., 2014) to express a genetically encoded pH sensor (synaptophysin pHluorin; SypHy) and/or light-activated proton pump (pHoenix; (Rost et al., 2015). We confirm, with SypHy post-stimulation fluorescence dynamics, that the pH profile of recycling GABAergic SVs differs from that of recycling glutamatergic SVs (Egashira et al., 2016). Using light-activation of pHoenix in pH-neutral vesicles, we investigated the pH dynamics of actively filling vesicles, and could show that proton efflux from GABAergic SVs is indeed initially faster than glutamatergic SVs in intact synapses. Finally, we compared the filling rate of empty glutamatergic and GABAergic vesicles using pHoenix as a proton source, and find a slightly faster filling of glutamatergic vs. GABAergic SVs.

## Introduction

In synaptic transmission, membrane bound synaptic vesicles (SVs) fuse with presynaptic plasma membrane, releasing their neurotransmitter content into the synaptic cleft. A crucial step in this process, therefore, is the packaging of neurotransmitter into the SVs by vesicular neurotransmitter transporters. At excitatory and inhibitory synapses in the CNS this is accomplished by the vesicular glutamate transporter (VGLUT) and vesicular GABA transporter (VGAT), respectively. Both of these transporters use the electrochemical force of a V-ATPase generated proton gradient to drive transport of their respective neurotransmitters, with the negatively charged glutamate thought to depend more heavily on the positive intraluminal SV membrane potential generated by the proton gradient and the neutral GABA dependent on both the electrical and chemical components of the proton gradient (Blakely and Edwards, 2012). The most recent estimates of transporter fill rate time constants from SVs in intact synapses is ~7 s for VGLUT (Hori and Takahashi, 2012) and ~25–40 s for VGAT (Egashira et al., 2016; Yamashita et al., 2018) at physiological temperature. However, details of their function, such as co- and countertransport ion identity and stoichiometry, have remained surprisingly enigmatic (Anne and Gasnier, 2014; Farsi et al., 2017). Understanding the biophysical properties of vesicular neurotransmitter transporters is important because these factors influence the rate of neurotransmitter uptake into SVs and may ultimately influence the availability of full SVs for release during repetitive stimulation of synapses.

Recent work has revealed differences in how SVs expressing VGLUT or VGAT proteins handle protons. At the whole synapse level, it has been confirmed that proton handling in GABAergic and glutamatergic vesicles results in differences in: (1) steady-state pH levels in respective vesicles/synapses; and (2) differences in the dynamics of pH during reacidification of recycled vesicles (Egashira et al., 2016). Isolated VGAT SVs also showed a higher steady state pH than isolated glutamatergic SVs, which was correlated with a faster proton efflux rate in GABAergic SVs (Farsi et al., 2016). Whether proton efflux from GABAergic and glutamatergic SVs in intact synapses differs and can be considered a mechanism for the differences in resting pH for the two SV populations is an open question.

To address the processes of SV refilling in glutamatergic and GABAergic synapses, we applied a glutamatergic- or GABAergic neuron-specific expression strategy (Chang et al., 2014) to express genetically encoded pH sensor and/or light-activated proton pump (pHoenix; Rost et al., 2015). This strategy resulted in a high throughput technique by which we could monitor SV recycling in glutamatergic and GABAergic synapses. With this technique, we could confirm that recycling GABAergic vesicles do indeed have a dynamic pH profile that differs from glutamatergic recycling vesicles (Egashira et al., 2016). By investigating the pH dynamics of actively filling vesicles using the SV-localized, light-activated proton pump, pHoenix, we could show that proton efflux from GABAergic vesicles is indeed initially faster than glutamatergic vesicles, and we suggest this due to a greater proton leak associated with the presence of VGAT, as previously shown (Farsi et al., 2016). This may reflect a difference in stoichiometry of proton exchange for neurotransmitter uptake in the respective vesicles. In addition, we could show that light activation of pHoenix, producing a proton gradient in pH-neutral vesicles, could support filling of SVs with either glutamate, as previously reported (Rost et al., 2015), or GABA. Finally, we use pHoenix-mediated recovery of postsynaptic responses in cultured glutamatergic or GABAergic neurons to compare the fill rate of SVs with neurotransmitter by VGLUT and VGAT, and find that glutamatergic SVs fill slightly faster than GABAergic SVs.

## Materials and Methods

### Neuronal Culture

All procedures were performed in accordance with national and institutional guidelines and approved by the Animal Welfare Committee of the Charité Universitätsmedizin and the health authority and ethics committee of the Berlin state government (Landesamt für Gesundheit und Soziales, Berlin; animal license number T0220/09).

Hippocampi or striata from VGAT-Cre or wildtype mice (P0–3) were removed and processed as previously described (Zimmermann et al., 2015). For fluorescence imaging experiments, dissociated VGAT-Cre hippocampal cells were plated on an astrocyte-layered glass coverslips at a density of approximately 130 cells/mm^2^. For autaptic electrophysiology experiments, wildtype dissociated hippocampal or striatal neurons were plated on astrocyte microdot islands (0.2 mm diameter) on 35 mm coverslips at a density of 3,500–4,000 cells/well. Experiments were performed on neurons at DIV 12–18.

### Lentivirus and Adeno-Associated Virus Constructs and Production

For cell-type-specific expression of synaptophysin pHluorin (SypHy; Granseth et al., 2006) or pHoenix (Rost et al., 2015), we used two different lentiviral vector constructs expressed in cultures where Cre expression is driven by the Viaat promoter (VGAT-Cre; Chao et al., 2010). As described in Chang et al. (2014), for expression in glutamatergic neurons, the sequence for the protein of interest controlled by the synapsin promoter was flanked by two LoxP sites, and Cre expression results in excision of the inserted sequence (“flox” vector). For expression in GABAergic neurons, the sequence for the protein of interest driven by the synapsin promoter was inserted in reverse orientation between two nested, incompatible pairs of Lox sites, LoxP and Lox2722. With this vector, Cre expression results in excision and inversion of the sequence, and thus protein expression only in Cre-positive cells (“SWITCH” vector). Expression of VGLUT2 in a non-cell-type-specific manner was achieved by a synapsin-promoter driven lentiviral construct. Cloning for all expression vectors were performed by the Charité Viral Core Facility (Charité—Universitätsmedizin Berlin).

Lentiviral particles for delivery of expression vectors to the neurons were prepared by the Charité Viral Core Facility as previously described (Lois et al., 2002). Briefly, HEK293T cells were cotransfected with the shuttle vector (10 μg) and helper plasmids, pCMVdR8.9 and pVSV.G (5 μg each) with polyethylenimine. Virus-containing supernatant was collected after 72 h, filtered, aliquoted, flash-frozen with liquid nitrogen, and stored at −80°C. For infection, virus was added to hippocampal neurons at 1 DIV. Adeno-associated virus (AAV) was prepared as described in Rost et al., 2015. Wildtype hippocampal or striatal autapses were infected with virus at DIV 1.

### Immunocytochemistry

Cultures (DIV 18–20) were washed with phosphate-buffered saline (PBS) solution, fixed with 4% paraformaldehyde, and processed as previously described (Chang et al., 2014). Primary antibodies were used as follows: rabbit anti-GFP (Abcam; Cat# Ab6556; 1:1,000 dilution); mouse anti-VGAT (Synaptic Systems; Cat# 131 011; 1:1,000 dilution); guinea pig anti-VGLUT1 (Synaptic Systems; Cat# 135 304; 1:4,000 dilution). Secondary antibodies were used as follows: Alex Fluor 488 donkey anti-rabbit (Jackson Immuno Research; Cat# 711-545-152; 1:500 dilution); Rhodamine Red donkey anti-guinea pig (Jackson Immuno Research; Cat# 706-295-148; 1:500 dilution); Alexa Fluor 647 donkey anti-mouse (Jackson Immuno Research; Cat# 715-605-151; 1:500 dilution).

Images were captured on an inverted microscope with 60× objective (water, n/a 1.2) using a CCD camera (Princeton Instruments) and mercury lamp illumination under control of MetaMorph Software (Molecular Devices). Colocalization of background-subtracted images was analyzed in ImageJ using the Pearson’s coefficient (*r*) of the full images determined by the JACoP colocalization plug-in (Bolte and Cordelières, 2006).

### Live Imaging Experiments

Live imaging of SypHy and pHoenix was performed on an inverted microscope (Olympus IX51, Olympus). Samples were perfused with an extracellular solution containing the following (in mM): 140 NaCl, 2.4 KCl, 10 HEPES (Merck), 10 glucose (Carl Roth), 4 CaCl_2_, (Sigma-Aldrich), 1 MgCl_2_ (Carl Roth); 300 mOsm; pH 7.4. Field stimulation was achieved by passing current between platinum electrodes of a field stimulation chamber (Warner Instruments). For all SypHy experiments involving stimulation, NBQX (5 μM), bicuculine (15 μM), and AP-5 (10 μM) were included in the perfusion solution. All experiments were performed at elevated temperature (~32–34°C) using simultaneously a custom-built in-line heating system and heated-collar for the objective (Warner Instruments).

Images were captured with a Hamamatsu ImagEM CCD camera under control of the Hamamatsu ImageLive software, using a 60× objective (1.2 n/a) and 2 × 2 binning. Samples were illuminated by light-emitted diode (LED; CoolLED, Prizmatics) at wavelength 470 nm for 100 ms (SypHy) or 50 ms (pHoenix), and captured at a rate of 0.5 Hz. Activation of pHoenix was performed using a dual filter system (AHF Analysentechnik, F56-019) with illumination of 470 nm wavelength (50 ms) interleaved with 1.5 s pulses of 550 nm wavelength LED during the activation period (2 min or 30 s light exposure).

Analysis of images was performed off-line using ImageJ (National Institutes of Health), Axograph X (Axograph), and Prism 5 (GraphPad). In ImageJ, image sequences were background-subtracted using the rolling-ball background subtraction (50 pixel) and aligned using a StackReg plug-in (Thévenaz et al., 1998) to correct for x-y drift when necessary. Regions of interest (ROIs) were selected from baseline-subtracted peak stimulation images (SypHy) or pHoenix-activation-subtracted baseline images (pHoenix). Values from image sequence ROIs measured in ImageJ were exported to Axograph and plotted as fluorescence (arbitrary units; a.u.) over time. ROIs were averaged per image and the average value of the image was treated as *n* = 1. Statistical analysis of values measured from average images was performed in Prism. The statistical test used, as indicated in figure legend, was determined based on number of groups analyzed and the normality of the values’ distribution (D’Agostino & Pearson normality test).

### Electrophysiology Experiments

Electrophysiological experiments at elevated temperature with perfusion of the extracellular solution as described for live imaging experiments. Whole-cell patch clamp recordings were made with borosilicate glass pipettes (3–4 MΩ) filled with an internal solution containing (in mM): 136 KCl, 17.8 HEPES, 1 EGTA (Carl Roth), 4.6 MgCl_2_, 4 Na_2_ATP, 0.3 Na_2_GTP (Sigma-Aldrich), 12 creatine phosphate (Calbiochem) and 50 U/ml phosphocreatine kinase (Sigma-Aldrich); 300 mOsm; pH 7.4. All recordings were performed with a Multiclamp 700B amplifier and Digidata 1440A under the control of Clampex 10 (all Molecular Devices). Data were acquired at 10 kHz and filtered at 3 kHz. Cells were recorded at a holding potential of −70 mV. For autapse recordings, series resistance was compensated to at least 60% and only cells with stable membrane properties for included for further analysis. For autapse recordings, pHoenix was activated by 550 nm wavelength through a DSRED filter (Olympus, U-N41035) and a 60× objective.

Analysis of electrophysiological data was performed using Axograph and Prism. Spontaneous postsynaptic currents (sPSCs) were detected using two different templates: for glu, amplitude −2 pA, rise time 0.5 ms, decay time 2 ms; for GABA, amplitude −2 pA, rise 2 ms, decay 10 ms. Strict detection parameters were employed by detecting events at five times standard deviation of the noise (uncompensated recordings, filtered *post hoc* at 1 kHz). Detected events were binned and averaged for amplitude, and frequency was calculated (count/bin length). Statistical analysis was performed using repeated measures ANOVA with Tukey’s Multiple Comparison test.

## Results

### Glutamatergic and GABAergic Synapses Display Differences in SV Reacidification Kinetics

**Figure 1:**
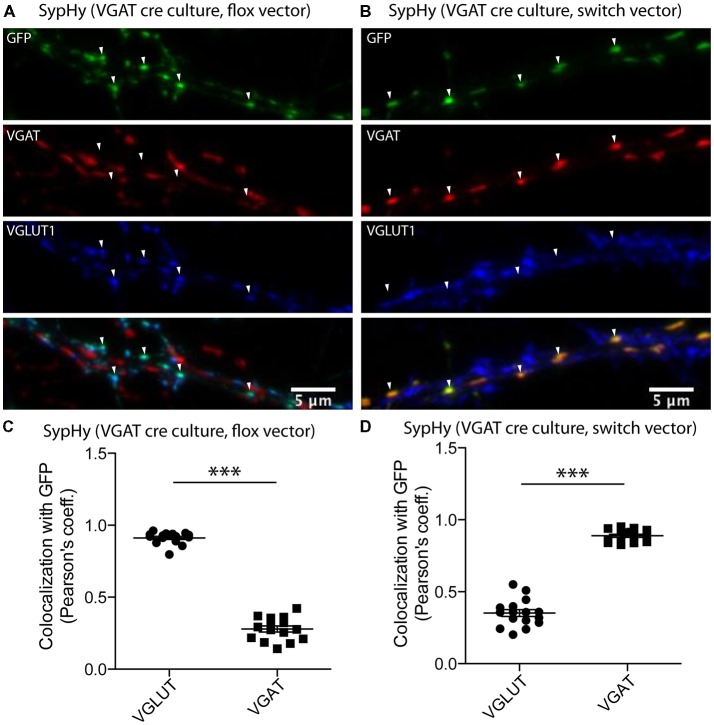
Synapse specific expression of synaptophysin pHluorin (SypHy) in vesicular GABA transporter (VGAT)-Cre culture synapses using flox/SWITCH expression vectors. Representative images of immunofluorescence from GFP (green, SypHy, vesicular glutamate transporter (VGLUT1; blue) and VGAT (red) in hippocampal VGAT-Cre cultures expressing SypHy by flox **(A)** or SWITCH **(B)** expression vectors. Arrows indicate GFP-positive regions of interest. **(C)** Colocalization of SypHy labeled with anti-GFP with either immunofluorescence for VGLUT1 or VGAT when SypHy is expressed by the flox expression vector in VGAT-cre hippocampal cultures. *n* = 15 images, three independent cultures. **(D)** Same as **(C)**, but with SypHy expressed by SWITCH vector. *n* = 16 images, three independent cultures. Colocalization determined by Pearson’s coefficient (r). Significance determined by paired *t*-test. ***p < 0.0001.

In both glutamatergic and GABAergic synapses, fusion of SVs with the plasma membrane is followed by recycling of membrane through endocytosis. During the recycling process, the acidic lumen of the SV is restored by V-ATPase and the resulting proton gradient drives refilling of SVs with neurotransmitter. However, the concept that the SV reacidification during recycling might vary depending on the vesicular neurotransmitter transporter present is new (Egashira et al., 2016). To assess the reacidification dynamics during SV recycling in glutamatergic and GABAergic synapses, it is necessary to monitor a pH reporter in the different synapse populations. To achieve this, we adapted a strategy using a two-vector system in cultures from the VGAT-Cre mouse line (Chang et al., 2014; see “Materials and Methods” section) to specifically express a genetically encoded pH sensor, SypHy in either glutamatergic or GABAergic synapses (Figure 1). In the VGAT-Cre cultures, expression of SypHy by the flox vector (see “Materials and Methods” section) should result in expression in Cre negative cells, while expression of SypHy with the SWITCH vector (see “Materials and Methods” section) should result in expression in Cre positive cells. To confirm that the Cre negative and positive expression corresponds to expression in glutamatergic and GABAergic synapses, respectively, we performed immunocytochemistry on VGAT-Cre cultures expressing SypHy by flox or SWITCH vectors. The SypHy signal, amplified by detection with a primary antibody against GFP, showed a significantly higher colocalization with VGLUT than VGAT when expressed by flox vector (Figure 1C) and vice versa when expressed by SWITCH vector (Figure 1D). These results, together with characterization of the technique from a previous study (Chang et al., 2014), suggest that VGAT-Cre combined with the flox/SWITCH expression vector system results in specific expression of proteins of interest in glutamatergic or GABAergic neurons.

**Figure 2:**
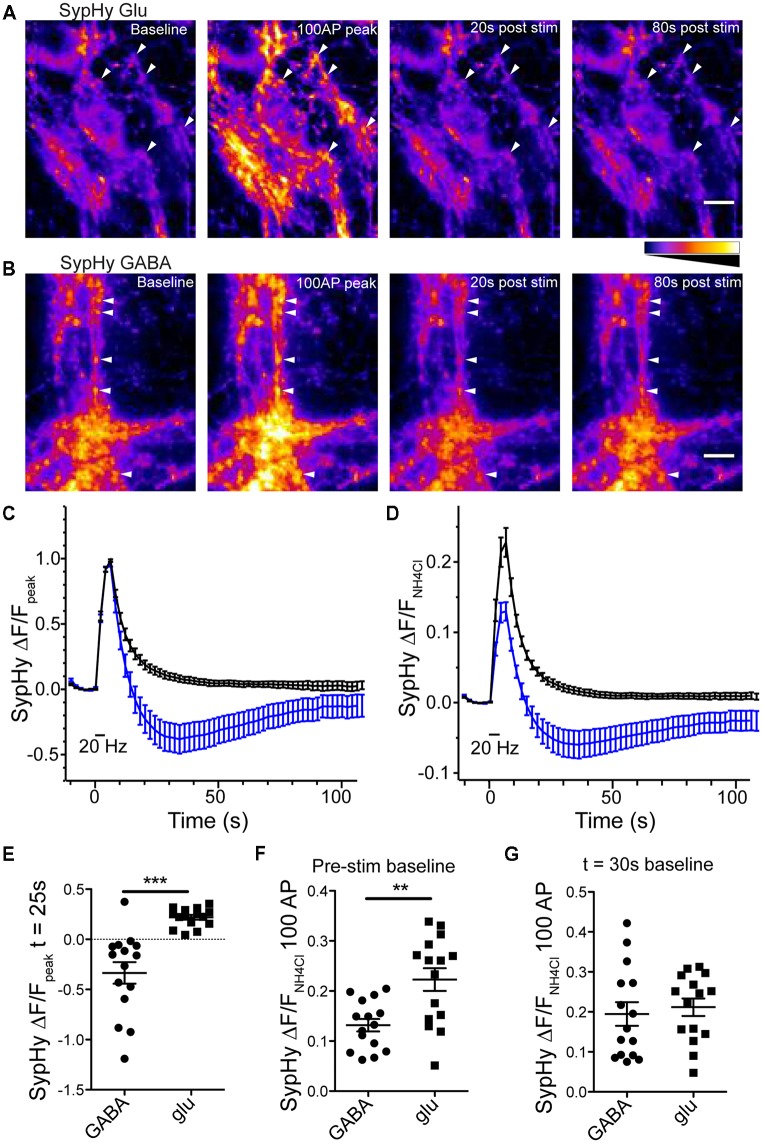
Reacidfication profile of SypHy differs in glutmatergic and GABAergic synapses. SypHy specifically expressed in glutmatergic **(A)** or GABAergic **(B)** synapses using flox or SWITCH expression vectors, respectively. Panels display SypHy fluorescence at baseline, immediately following a 100 pulse, 20 Hz stimulus, and 20 or 80 s post-stimulus. All panels set to intensity scale 0–2500 a.u. Scale bar = 10 μm. Arrows indicate select regions of interest (ROIs). For analysis, the baseline fluorescence for each image was subtracted from the 100 AP image (100 AP ΔF). ROIs were selected from the 100 AP ΔF. All ROIs for a given image were measured as fluorescence over time and averaged to give a single fluorescence trace for each image. **(C,D)** Average change in SypHy fluorescence over timenormalized to the peak fluorescence after a 100 pulse, 20 Hz stimulus **(C)** or normalized to the peak fluorescence in response to a 50 mM ammonium chloride solution **(D)** in glutamatergic (black traces; *n* = 15 images; four independent cultures) or GABAergic (blue traces; *n* = 15 images; four independent cultures) synapses. Mean ± SEM. **(E)** Scatter plot of SypHy fluorescence normalized to 100 pulse, 20 Hz stimulus at 25 s post-stimulus. Significance determined by unpaired *t*-test, ***p < 0.0001. **(F,G)** Comparison of SypHy fluorescence response to 100 pulse, 20 Hz stimulus normalized to 50 mM ammonium chloride when profiles were zeroed at the pre-stimulus fluorescence **(F)** or fluorescence at the time point 30 s post-stimulation **(G)**. Bars = Mean ± SEM. Significance determined by unpaired *t*-test. **p = 0.0015.

Expression of SypHy in glutamatergic or GABAergic synapses resulted in a sharp increase in fluorescence in response to a 100 pulse field stimulation at 20 Hz, indicative of SV exocytosis and SypHy exposure to pH neutral extracellular environment, followed by a fluorescence decay, indicative of endocytosis of SypHy and reacidification of the endocytosed structures (Figures 2A,B). However, as reported by Egashira et al. (2016) in GABAergic synapses, the SypHy fluorescence decay normalized to the peak fluorescence increase dipped below the baseline fluorescence and slowly increased back to baseline, while glutamatergic synapses exhibited a standard SypHy decay (Atluri and Ryan, 2006; Granseth et al., 2006; Figures 2A–E). When the SypHy profiles were normalized to a pulse of ammonium chloride (NH_4_Cl, 50 mM) to alkalinize all intraluminal SypHy, or the entire vesicle pool, the average amplitude of fluorescence change in response to a 100 action potential (AP) stimulation at 20 Hz was significantly smaller in GABAergic synapses than in glutamatergic synapses (Figures 2D,F). However, if the NH_4_Cl normalized traces were set to baseline at the lowest point on average of the SypHy decay (25 s after stimulation ended) the peak response to 100 AP stimulation was not different between the two synapse types (Figure 2G). Taken together, these data suggest that GABAergic vesicles acidify below their resting intraluminal pH after stimulation, and return to their steady-state pH over time. Though we did not test it directly, these results are consistent with previous findings using the pH sensor variant, mOrange, suggesting that GABAergic vesicles have a higher resting pH than glutamatergic vesicles (Egashira et al., 2016).

**Figure 3:**
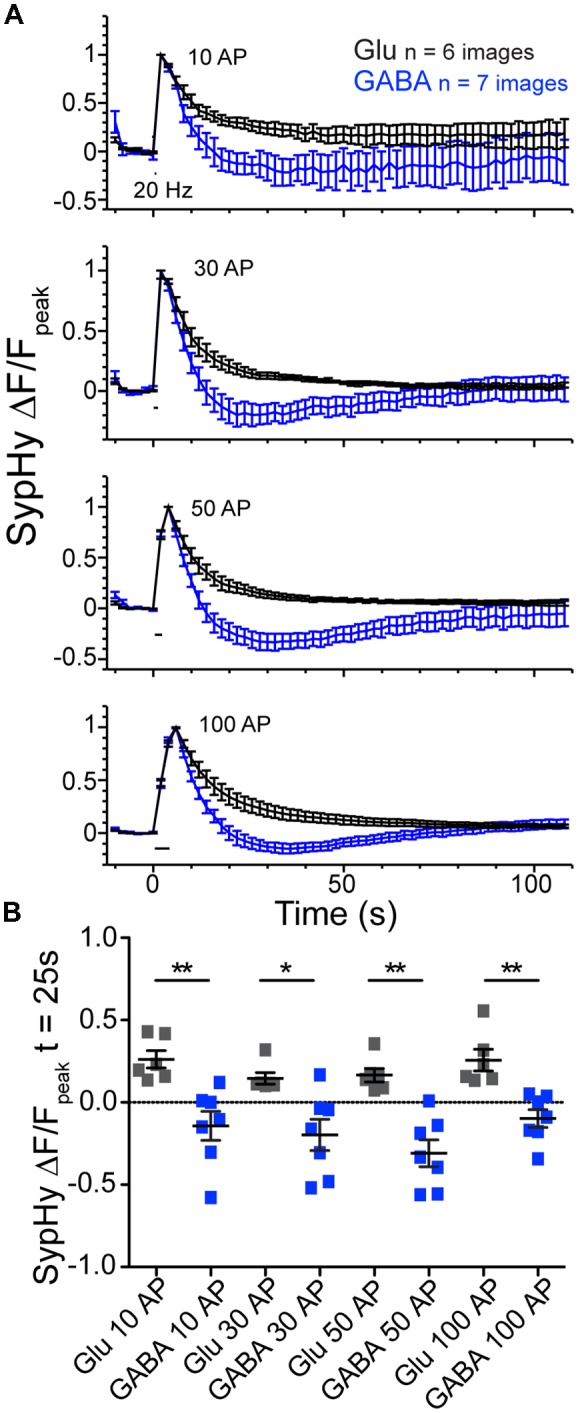
Glutamatergic and GABAergic synaptic vesicles (SVs) display differences in reacidification processes regardless of stimulus number. **(A)** Average SypHy fluorescence normalized to peak fluorescence after stimulation in glutamatergic (black) or GABAergic (blue) synapses stimulated with 10, 30, 50 and 100 pulses at 20 Hz. Stimulation duration (20 Hz) indicated by black interval bar. Mean ± SEM, three independent cultures. **(B)** Scatter plot of SypHy fluorescence normalized to the peak fluorescence for each stimulation train at a time point 25 s post stimulation. Bars = Mean ± SEM. Significance determined by Mann Whitney test. 10 AP, **p = 0.0012; 30 AP, *p = 0.0221; 50 AP, **p = 0.0012; 100 AP, **p = 0.0012.

To further probe the phenomenon of GABAergic vesicle acidification below baseline after stimulation, we perturbed the standard stimulation conditions. First, we investigated whether the pH dip occurred if the stimulation train contained fewer stimuli (Figure 3). We saw that in addition to the 100 AP stimulation, the difference between the SypHy decay time courses in GABAergic and glutamatergic synapses was also significant with 10, 30 and 50 pulse trains at 20 Hz (Figures 3A,B). Second, we found that introducing a VGLUT transporter onto GABAergic vesicles, by overexpression of exogenous VGLUT2, did not significantly alter the kinetics of their SypHy fluorescence decay (data not shown; ΔF/F_peak_ at *t* = 25 s, average ± SEM, GABAergic control −0.21 ± 0.08, *n* = 16; +VGLUT2 −0.09 ± 0.08, *n* = 14; *p* = 0.301, Mann-Whitney test), even though we have shown previously that VGLUT2 overexpression introduces glutamate flux into single GABAergic vesicles (Zimmermann et al., 2015). This suggests that the presence of intraluminal glutamate alone does not account for the difference in SypHy fluorescence decay kinetics after stimulation in glutamatergic and GABAergic synapses.

### Light-Induced Acidification of Glutamatergic and GABAergic SVs Using Specific Expression of pHoenix

**Figure 4:**
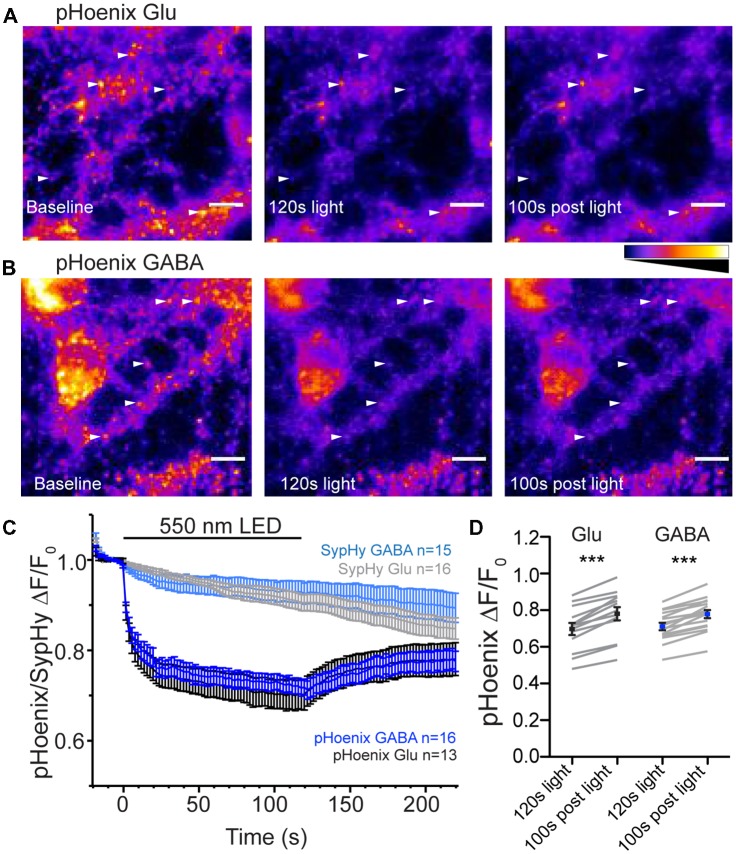
pHoenix acidifies glutmatergic and GABAergic SVs in intact synapses. **(A,B)** Images of bafilomycin-treated (>2 h, 1 μm) cultures expressing pHoenix in glutamatergic **(A)** or GABAergic **(B)** synapses at baseline, after activation of pHoenix with 550 nm LED for 120 s, and a time point 100 s after end of light stimulation. Image intensity scales for all panels were set to 0–1300 a.u. **(A)** or 0–700 a.u. **(B)**. Arrows indicate particular ROIs. Scale bar = 10 μm. **(C)** Average fluorescence response normalized to baseline fluorescence in bafilomycin-treated cultures expressing pHoenix in glutamatergic (black; *n* = 13 images; three independent cultures) or GABAergic (blue; *n* = 16 images; three independent cultures) synapses or expressing only SypHy in glutamatergic (gray; *n* = 16 images; two independent cultures) or GABAergic (light blue; *n* = 15 images; two independent cultures) stimulated with 550 nm LED for 120 s. The SypHy-only images are included for control purposes and were performed in a separate set of experiments. **(D)** Comparison of pHoenix fluorescence normalized to baseline in bafilomycin-treated cultures at time points immediately after 120 s, 550 nm light stimulation and 100 s after completion of light stimulation in glutamatergic (black) and GABAergic (blue) synapses. Mean ± SEM. Significance determined by paired *t*-test. Glu ***p < 0.0001; GABA, ***p < 0.0001.

The mechanism determining the SypHy decay profile at GABAergic synapses is proposed to be a less acidic pH in the GABAergic vesicles under steady-state conditions, mediated by the presence of the VGAT protein (Egashira et al., 2016). Another recent study suggests that isolated GABAergic SVs have a faster proton efflux rate than isolated glutamatergic SVs (Farsi et al., 2016). It is not clear whether this difference in proton efflux between the two vesicles types is preserved in intact synapses. To address this, we used a genetically-encoded, exogenous proton source, pHoenix (Rost et al., 2015), specifically expressed in GABAergic or glutamatergic synapses using the SWITCH or flox vectors, respectively, in VGAT-Cre hippocampal cultures (Figure 4). pHoenix is a chimera protein in which the bacterial light-activated proton pump, Arch3 is conjugated to synaptophysin and pHluorin, leading to an SV localized, light-activated proton pump with a built-in pH sensor (Rost et al., 2015). To gauge whether pHoenix functions as expected with our expression system, i.e., can generate a proton gradient capable of supporting uptake of neurotransmitter by vesicular transporters, we first treated the VGAT Cre cultures expressing pHoenix with V-ATPase inhibitor, bafilomycin A1 (baf), for 2–5 h. Inhibition of the V-ATPase with baf abolishes the ability to establish a proton gradient, leaving SVs at a neutral pH and rendering them unable to be refilled with neurotransmitter (Zhou et al., 2000). In the baf-treated cultures, green fluorescence from the pHluorin pH sensor in pHoenix was visible in glutamatergic (Figure 4A) and GABAergic (Figure 4B) synapses, suggesting that baf-treated vesicles were pH-neutral as expected. The fluorescence decreased upon exposure of the synapses to 550 nm wavelength light for 2 min, quenching to approximately 70% of baseline in both glutamatergic and GABAergic synapses (*p* = 0.7421, unpaired *t*-test; Figures 4C,D), suggesting that the activation of the proton pump in pHoenix by light resulted in translocation of protons into the SVs, resulting in acidification of the SV lumen and quenching of the pHoenix pH probe.

The initial characterization of pHoenix showed that the rapid decrease in fluorescence of the pH sensor in pHoenix in response to red-shifted light was due to presence of the proton pump and not bleaching of the pHluorin itself (Rost et al., 2015). Nevertheless, as we used a wavelength of 550 nm to activate pHoenix (vs 580 nm in Rost et al., 2015), we repeated the control experiments using our conditions. In a separate set of experiments, we expressed SypHy alone (in the absence of the light-activated proton pump) in glutamatergic or GABAergic neurons. The baseline fluorescence in baf-treated cultures expressing SypHy in glutamatergic or GABAergic synapses was not rapidly quenched by exposure to 550 nm light (Figure 4C). These results are consistent with the original characterization of pHoenix (Rost et al., 2015).

Interestingly, in the case of fluorescence of the pHoenix pH sensor, there was a slight, but highly significant rebound in the fluorescence back towards baseline after cessation of the light pulse in both synapse types (Figure 4D), suggesting an escape of protons. This is in contrast to the stable pH in vesicles reported after pHoenix activation in glutamatergic autapses (Rost et al., 2015); however, this difference is likely due the elevated temperature conditions of the current experiments.

### Faster Proton Efflux From GABAergic SVs in Intact Synapses

**Figure 5:**
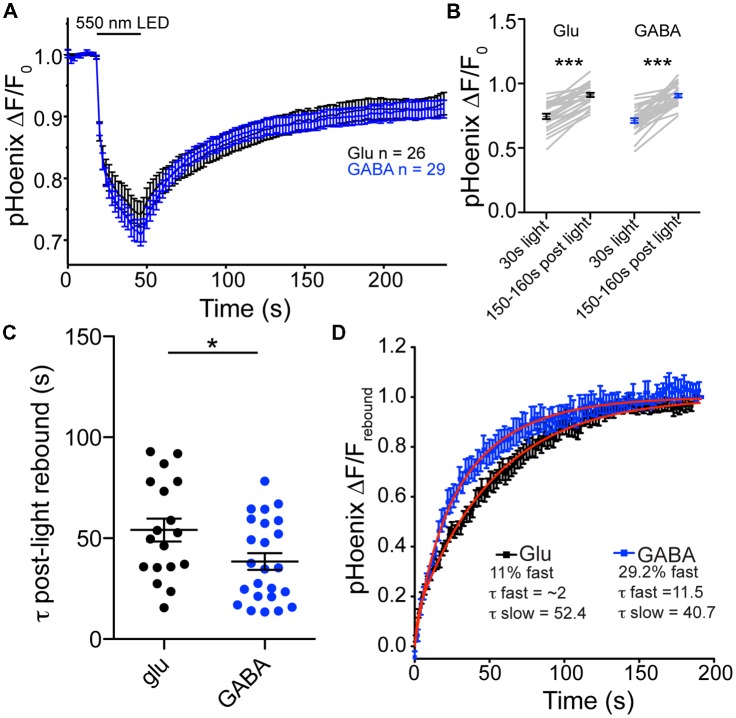
Proton efflux is faster from GABAergic SVs than glutamatergic SVs after pHoenix-induced acidification. **(A)** Average decrease in pHoenix fluorescence normalized to baseline fluorescence with 550 nm light stimulation (30 s) in VGAT-cre cultures pre-treated with bafilomycin (>2 h, 1 μM) in glutamatergic (black; *n* = 26 images; four independent cultures) or GABAergic (blue; *n* = 29 images; four independent cultures) synapses. **(B)** Comparison of normalized pHoenix fluorescence at time points immediately following a 30 s stimulation with 550 nm light and 150–160 s after light cessation in glutamatergic (black) and GABAergic (blue) synapses. Significance determined by paired *t*-test. ***p < 0.0001. **(C)** Scatter plot of τ calculated from single or double exponential fit (as determined by best fit) of rebound fluorescence after 30 s stimulation of pHoenix by 550 nm light in bafilomycin pre-treated VGAT cre cultures. Bars = Mean ± SEM. Significance determined by unpaired *t*-test. *p = 0.0294. **(D)** Double exponential fit (red lines) to average rebound fluorescence set to baseline immediately after a 30 s stimulation with 550 nm light and normalized to peak rebound fluorescence 150–160 s post light per image in glutamatergic (black) and GABAergic (blue) synapses. Mean ± SEM.

Is there a difference in the rate of proton efflux from glutamatergic and GABAergic SVs in intact synapses? To assess the issue of proton efflux, we again turned to specific expression of pHoenix in glutamatergic and GABAergic synapses. In bafilomycin-treated cultures, we chose a light application time of 30 s, so that SVs would be acidified, but not full (Rost et al., 2015). During the 30 s light pulse, the baseline fluorescence in baf-treated cultures was reduced to similar extents in glutamatergic or GABAergic synapses (74.5% or 71.2% of baseline fluorescence, respectively; *p* = 0.2347, unpaired *t*-test; Figures 4A,B). In the post-light period, the fluorescence rebounded significantly in both glutamatergic and GABAergic synapses, presumably due to loss of protons from the SVs, be it specific or non-specific (Figures 5A,B). The rebound fluorescence reached the same plateau value with regard to the baseline fluorescence in GABAergic and glutamatergic synapses (Figures 5A,B). However, we found that the fluorescence rebound after cessation of the light pulse in GABAergic synapses occurred with a faster time course than that in glutamatergic synapses (Figure 5C; only fits with time constants less than 95 s were included for analysis). In individual experiments, 46% of GABAergic images showed a two-exponential rise, compared with only 28% of glutamatergic images. In addition, the average tau to reach the plateau was significantly faster in GABAergic synapse images (Figure 5C). A fit to the average rebound of all images revealed a two-exponential rise (Figure 5D) where the fast component was 29.2% in GABAergic synapses (τ_fast_ = 11.5 s, τ_slow_ = 40.7 s) and 11% in glutamatergic synapses (τ_fast_ = ~2 s, τ_slow_ = 52.4 s). Taken together, these data suggest that proton efflux is indeed faster in GABAergic SVs even in intact synapses, in turn, suggesting that increased proton efflux is responsible for the higher steady-state pH in GABAergic SVs.

### Neurotransmitter Uptake Driven by pHoenix-Mediated Light-Activated Proton Gradient

**Figure 6:**
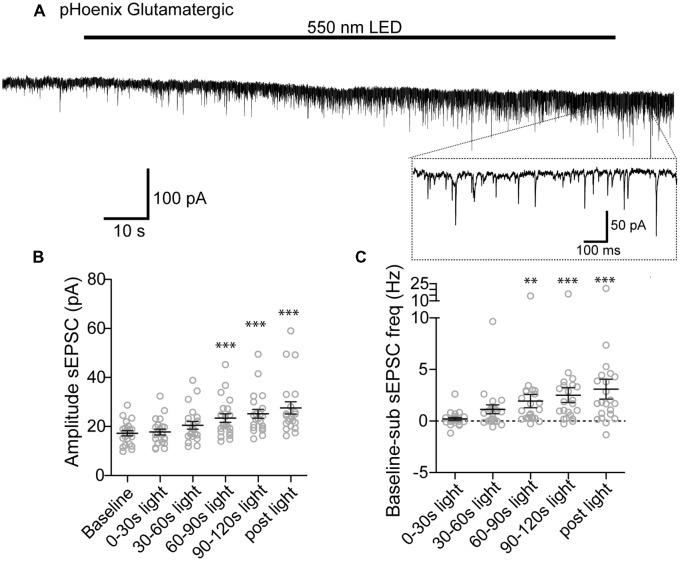
pHoenix-generated acidification drives glutamate uptake in glutamatergic SVs. **(A)** Example recording of bafilomycin-treated (>2 h, 1 μM) VGAT-cre culture expressing pHoenix in glutamatergic synapses, stimulated with 550 nm LED for 120 s. Scatter plot of average amplitude **(B)** and frequency **(C)** per cell of spontaneous excitatory postsynaptic currents (sEPSCs) detected during the baseline period (18 s), and 0–30 s, 30–60 s, 60–90 s, 90–120 s 550 nm light, and immediately following cessation of light stimulation (12 s). *n* = 21 cells. Two independent cultures Recordings were made in VGAT-cre cultures pretreated with bafilomycin and expressing pHoenix in glutamatergic synapses. Bars = Mean ± SEM. Significance was determined by repeated-measures ANOVA with Tukey’s multiple comparison test. ***p < 0.001. **p < 0.01.

**Figure 7:**
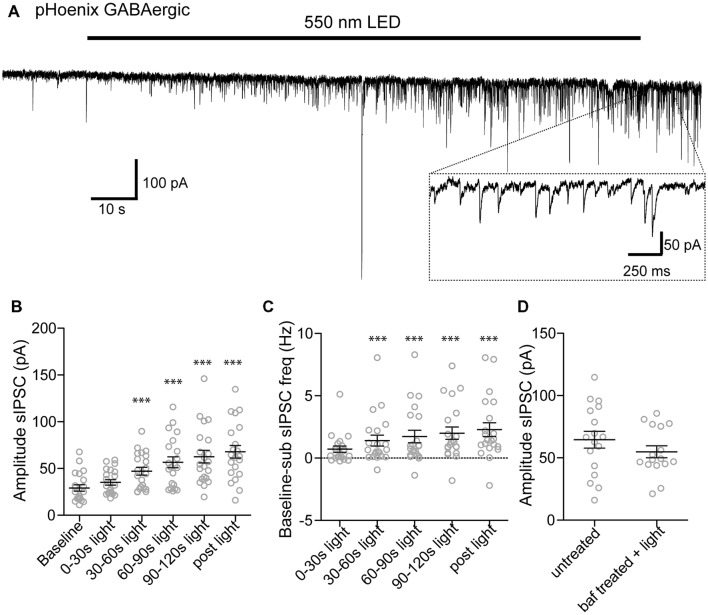
pHoenix-generated acidification drives GABA uptake in GABAergic SVs. **(A)** Example recording of bafilomycin-treated (>2 hs, 1 μM) VGAT-cre culture expressing pHoenix in GABAergic synapses, stimulated with 550 nm LED for 120 s. Scatter plot of average amplitude **(B)** and frequency **(C)** per cell of spontaneous inhibitory postsynaptic currents (sIPSCs) detected during the baseline period (18 s), and 0–30 s, 30–60 s, 60–90 s, 90–120 s 550 nm light, and immediately following cessation of light stimulation (12 s). Recordings were made in VGAT-cre cultures pretreated with bafilomycin and expressing pHoenix in GABAergic synapses. *n* = 21 cells. Two independent cultures. Bars = Mean ± SEM. Significance was determined by repeated-measures ANOVA with Tukey’s multiple comparison test. ***p < 0.001. **(D)** Scatter plot of sIPSCs recorded from untreated VGAT-cre cultures in the presence of NBQX (10 μM) and those evoked in bafilomycin pre-treated cultures with GABAergic neuron-expressed pHoenix activated by 550 nm light for 2 min. Bars = Mean ± SEM. Significance determined by unpaired *t*-test. *p* = 0.26. Two independent cultures.

We next tested whether pHoenix induced acidification of SVs can drive neurotransmitter uptake of glutamate and GABA in our system. After disruption of the proton gradient source, SVs that have been released and recycled cannot be refilled, and are therefore empty. Therefore, to observe neurotransmitter filling driven by a pHoenix-mediated proton gradient, we performed whole-cell voltage-clamp experiments in the baf-treated VGAT-Cre cultures, and activated pHoenix with the 2 min 550 nm wavelength light pulse. We found that during the light application, spontaneous postsynaptic currents (sPSCs) with kinetics reflecting glutamatergic (AMPA; Figure 6A) or GABAergic receptors (Figure 7A) emerged in cultures where pHoenix expression was driven by the flox or SWITCH vectors, respectively. In glutamatergic synapses, both the amplitude and frequency of sPSCs significantly increased above baseline after 60–90 s of pHoenix activation with 550 nm wavelength light (Figures 6B,C). In GABAergic synapses, both the amplitude and frequency of sPSCs significantly increased above baseline after 30–60 s of pHoenix activation (Figure 7B,C). However, we suggest that the slightly faster emergence of GABAergic sPSCs is due to their relatively larger size and slower kinetics, thus allowing easier detection above baseline noise.

As filling of GABAergic vesicles by pHoenix has not previously been shown, we tested the efficacy the pHoenix-based proton source for loading GABA into vesicles as compared to the endogenous, V-ATPase proton source. To this end, we recorded sPSCs in the presence of AMPAR antagonist, NBQX, from cultures expressing pHoenix in GABAergic synapses without baf treatment and after 2–5 h baf treatment plus 2 min 550 nm wavelength light. We found no difference in the amplitude of GABAR-mediated sPSCs in either condition (Figure 7D), suggesting that pHoenix-mediated acidification is sufficient to fully fill SVs with GABA.

### Analysis of Transport Rates for Glutamate and GABA Uptake Into SVs Using a pHoenix-Generated Proton Gradient

**Figure 8:**
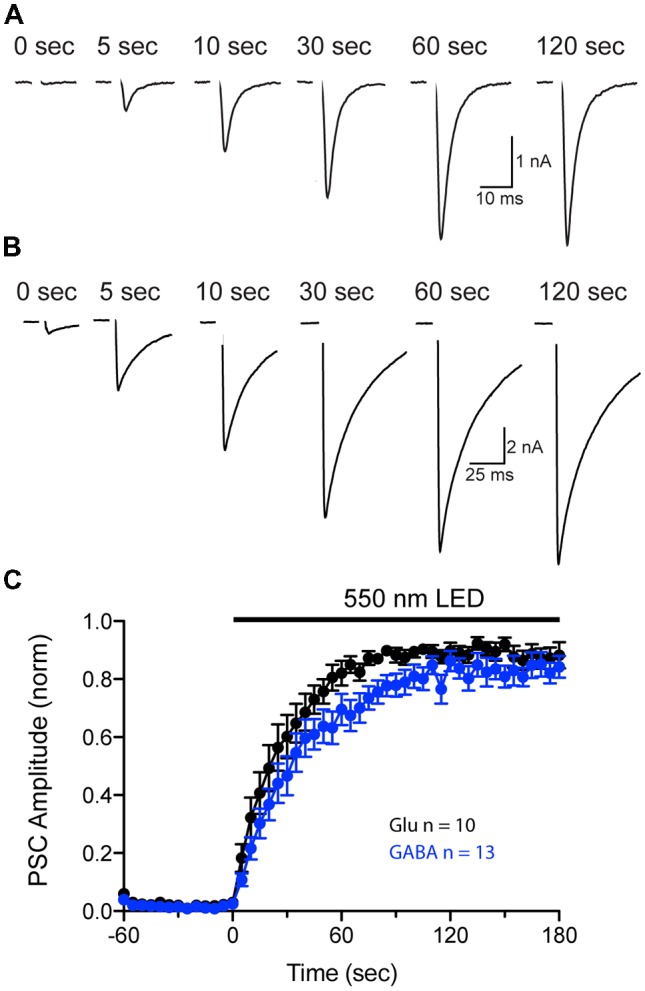
Comparison of pHoenix-driven Postsynaptic current (PSC) recovery in glutamatergic and GABAergic autapses. **(A,B)** PSCs recorded from a representative bafilomycin-treated glutamatergic **(A)** or GABAergic **(B)** autaptic cultured neuron expressing pHoenix at indicated time points during a 120 s exposure to 550 nm light. Depolarization-evoked, unclamped action potential has been blanked for illustrative purposes. **(C)** Average recovery time course of PSCs from bafilomycin-treated, pHoenix-expressing glutamatergic (black; *n* = 10 cells; two independent cultures) and GABAergic (blue; *n* = 13 cells; two independent cultures) autaptic neurons. For each cell, baseline PSC amplitudes (60 s pre-light exposure) were subtracted then normalized to the peak response during a 180 s exposure to 550 nm light.

Our previous experiments show that pHoenix activation results in both acidification and filling of both VGLUT and VGAT expressing SVs (Figures 4–7). However, comparison of the neurotransmitter fill rate is complicated by differences in the kinetics of sPSCs generated by postsynaptic AMPA and GABA receptors. Therefore, to further investigate whether a pHoenix-generated proton gradient can drive uptake of glutamate and GABA at a similar rate, we turned to analysis of postsynaptic current (PSC) recovery after baf-treatment in autaptic cultures (Figure 8). Wildtype autapses cultured from hippocampus or striatum were induced to express pHoenix by AAV infection (Rost et al., 2015) and were treated with baf as previously described for the VGAT-cre mass cultures. Under whole cell voltage-clamp, baf-treated autaptic neurons were induced to elicit an unclamped action potential by brief depolarization (1 ms), and PSCs were monitored before and during exposure to 550 nm light. Activation of pHoenix by 550 nm light in baf-treated autaptic neurons resulted in an increased glutamatergic (Figure 8A) or GABAergic (Figure 8B) PSC. On average, pre-light, baf-treated baseline responses in glutamatergic autapses (0.09 ± 0.07 nA) were significantly increased to 5.93 ± 1.48 nA (*n* = 11 cells; **p = 0.0031; paired *t*-test; Mean ± SEM) at 2–3 min of 550 nm light, while baseline GABAergic responses (0.94 ± 0.54 nA) significantly increased to 9.77 ± 1.92 nA (*n* = 14 cells; ***p = 0.0002; paired *t*-test; Mean ± SEM). The average recovery time course of PSCs normalized per cell to the peak PSC during light application could be fitted by a single exponential for both glutamatergic and GABAergic autapses (Figure 8C). One cell was excluded from each group as light did not elicit a PSC response. Fitting of averaged data yielded a slightly faster time constant from glutamatergic vs. GABAergic autapses (26.5 s and 36.3 s, respectively).

## Discussion

In chemical synaptic transmission, packaging of neurotransmitters into membrane-bound vesicles destined for fusion with the plasma membrane is a crucial step. The vesicular neurotransmitter transporters that accomplish this task use a driving force generated by the same source, the V-ATPase, regardless of the neurotransmitter type. Our work supports the idea that the electrochemical driving force for neurotransmitter transporters, the V-ATPase-generated proton gradient is used in a slightly different manner depending on the identity of the transmitter being transported. We find, in agreement with previously published findings (Egashira et al., 2016), that reacidification dynamics of GABAergic vesicles differs from that in glutamatergic vesicles. We show that proton loss from VGAT containing, GABAergic SVs occurs with a faster rate in intact synapses. This is also in good agreement with recent findings from isolated VGAT and VGLUT containing vesicles (Farsi et al., 2016), and serves as a nice validation for processes observed in isolated SVs occurring in intact synapses. Finally, we show that an exogenous proton source can support neurotransmitter uptake by both VGLUT and VGAT, and we compare the uptake rate for glutamate and GABA in small central synapses.

### High-Throughput Cell-Type Specific Assay of SV pH Dynamics

Until recently, the processes of endocytosis and SV recycling at presynaptic terminals have largely been generalized between inhibitory and excitatory neurons. Specific analysis of genetically encoded probes, such as pH sensitive fluorescent proteins, expressed in synapses of known cellular identity is a crucial step to comparing synaptic biology between cell types. The differences in pH dynamics in glutamatergic and GABAergic SVs was originally reported by using colocalization of pH sensitive sensors with a second fluorescent protein specifically expressed in GABAergic synapses (Egashira et al., 2016). In our current experiments, we show that specific expression of the pH sensor itself, SypHy, in glutamatergic or GABAergic synapses can reproduce the previously reported results. With our flox/SWITCH vector system expressed in VGAT-driven Cre-expressing primary cultures, we could compare in a high-throughput manner reacidification dynamics of recycling vesicles using SypHy, SV filling with an exogenous proton source, and proton efflux from SVs between glutamatergic and GABAergic synapses. Beyond our results, this technique has promise for investigating synaptic properties using genetically encoded tools in a precise manner defined by the cell-type specificity of Cre expression.

### Potential Causes for and Implications of Increased Proton Efflux From VGAT SVs

In addition to synapse-specific expression of SypHy, we also used the VGAT-Cre driven SWITCH/flox expression system to examine VGAT and VGLUT function with an exogenous proton source, the SV-localized, light-activated proton pump, pHoenix. By monitoring the internal pHluorin probe, we saw that the pH rebound after a 30 s activation pulse of pHoenix was faster in VGAT vs. VGLUT vesicles (Figure 5). Our interpretation of this phenomenon is that VGAT vesicles experience a faster proton efflux rate, as observed in isolated, VGAT SVs (Farsi et al., 2016). Why VGAT SVs have a faster proton efflux rate is an open question. One intriguing possibility could be a difference in proton stoichiometry per transport cycle. As of yet, though both VGLUT (Farsi et al., 2016; Martineau et al., 2017; but see Eriksen et al., 2016) and VGAT (Farsi et al., 2016) are suggested to use protons as antiport for neurotransmitter uptake, the exact stoichiometry is unknown (Edwards, 2007; Anne and Gasnier, 2014; Farsi et al., 2017). Therefore, the possibility exists that VGAT uses two protons per GABA molecule as countertransport, while VGLUT uses only one per glutamate molecule. As GABA is converted from glutamate by glutamate decarboxylase, presumably the cytosolic GABA concentration is lower than the cytosolic glutamate concentration. From an energetics standpoint, two protons as countertransport could provide a higher driving force to concentrate GABA against a steeper concentration gradient. This could function in an analogous to the plasma membrane glutamate transporter, which harnesses the energy of three Na^+^ ions for co-transport of a glutamate molecule, allowing it to work against a very steep concentration gradient and keep the extracellular glutamate concentration extremely low (Tzingounis and Wadiche, 2007). Future work is necessary to further address the biophysical details of how these vesicular transporters concentrate neurotransmitter molecules in the SVs.

However, while our results for the fluorescence rebound after 30 s of pHoenix activation in glutamatergic and GABAergic SVs are consistent with the interpretation of a faster proton efflux as observed in isolated vesicles, it cannot be ruled out that the pHoenix localization is different in the two synapse types, and that in GABAergic synapses more probe is localized to other compartments that are leakier for protons. For instance, in its initial characterization the rebound fluorescence when pHoenix was localized to lysosomes was much faster than when localized to SVs (Rost et al., 2015). While it is clear from our experiments that pHoenix is localized to SVs, because we get a recovery of GABAergic responses when the endogenous proton gradient is destroyed, it cannot be ruled out that some fraction of pHoenix is localized to lysosomal compartments.

### Exogenous Proton Source Generates Uptake by VGLUT and VGAT

Previous work showed that a pHoenix-generated proton gradient can support glutamate uptake into SV vesicles in glutamatergic neurons cultured as isolated autapses (Rost et al., 2015). Our work expands these findings to show that light-activated acidification of SVs by pHoenix can also support GABA uptake in VGAT-containing SVs of GABAergic terminals, and fills GABAergic SVs to a similar level as the endogenous proton pump (Figure 7).

In addition, we have gone on to use pHoenix to compare the uptake rate of glutamate and GABA in small central synapses at near-physiological temperature. Previous work has shown that with the endogenous proton source, the time constant for refilling of empty vesicles with uncaging of glutamate at 37°C is around 7 s in the calyx of Held (Hori and Takahashi, 2012). In contrast, the time constant reported for filling of GABAergic vesicles is between 25–40 s (Egashira et al., 2016; Yamashita et al., 2018). We sought to compare the fill rates of vesicles for glutamate and GABA within the same preparation by using bafilomycin treatment and pHoenix-mediated recovery of PSCs in wildtype glutamatergic and GABAergic autapses. Interestingly, we found that the time course of the averaged PSC recoveries from GABAergic autapses was in the range as that reported for GABAergic synapses by pHluorin extrapolation (Egashira et al., 2016) and by GABA uncaging in the terminals of inhibitory neurons (Yamashita et al., 2018). However, while the filling time course estimate for glutamatergic autapses was slightly faster than GABAergic autapses, it was still more than three-fold slower than time course estimated from calyx of Held (Hori and Takahashi, 2012). The discrepancy in these values may have a number of sources. One possibility is that the efficiency of the exogenous proton source, pHoenix, may not reach that of the endogenous V-ATPase, causing the pHoenix proton pump to be rate-limiting. A second possibility is that there are biological differences between glutamatergic SVs in small hippocampal nerve terminals and at the calyx of Held. For instance, it was determined by Hori and Takahashi (2012), that SVs in immature calyces have a significantly slower fill rate than those of mature calyces. The possibility exists that the biological composition of SVs in our cultured glutamatergic autapse may also differ from the glutamatergic SVs from calyx of Held synapses in acute brain slice.

Finally, the combination of pHoenix expression in cultured autapses with genetic manipulation could provide a unique tool for investigating more subtle effects on vesicular neurotransmitter function than has previously been possible. For example, comparison of fill rates in autapses expressing one or two copies of the transporter gene (wildtype vs. heterogenous) or comparison of wildtype or point-mutant transporters in autapses with a knockout background will provide important insights into how vesicular neurotransmitter transporters function.

## Author Contributions

MH performed research and analyzed data and wrote the article. MH and CR designed research and interpreted results. TT provided molecular biology tools.

## Conflict of Interest Statement

The authors declare that the research was conducted in the absence of any commercial or financial relationships that could be construed as a potential conflict of interest.

## References

[B1] Anne C. Gasnier B. (2014). Vesicular neurotransmitter transporters: mechanistic aspects. Curr. Top. Membr. 73, 149–174. 10.1016/B978-0-12-800223-0.00003-7 24745982

[B2] Atluri P. P. Ryan T. A. (2006). The kinetics of synaptic vesicle reacidification at hippocampal nerve terminals. J. Neurosci. 26, 2313–2320. 10.1523/JNEUROSCI.4425-05.2006 16495458 PMC6674811

[B3] Blakely R. D. Edwards R. H. (2012). Vesicular and plasma membrane transporters for neurotransmitters. Cold Spring Harb. Perspect. Biol. 4:a005595. 10.1101/cshperspect.a005595 22199021 PMC3281572

[B4] Bolte S. Cordelières F. P. (2006). A guided tour into subcellular colocalization analysis in light microscopy. J. Microsc. 224, 213–232. 10.1111/j.1365-2818.2006.01706.x 17210054

[B5] Chang C.-L. Trimbuch T. Chao H.-T. Jordan J.-C. Herman M. A. Rosenmund C. (2014). Investigation of synapse formation and function in a glutamatergic-GABAergic two-neuron microcircuit. J. Neurosci. 34, 855–868. 10.1523/jneurosci.0229-13.2014 24431444 PMC6608345

[B6] Chao H.-T. Chen H. Samaco R. C. Xue M. Chahrour M. Yoo J. (2010). Dysfunction in GABA signalling mediates autism-like stereotypies and rett syndrome phenotypes. Nature 468, 263–269. 10.1038/nature09582 21068835 PMC3057962

[B7] Edwards R. H. (2007). The neurotransmitter cycle and quantal size. Neuron 55, 835–858. 10.1016/j.neuron.2007.09.001 17880890

[B8] Egashira Y. Takase M. Watanabe S. Ishida J. Fukamizu A. Kaneko R. (2016). Unique pH dynamics in GABAergic synaptic vesicles illuminates the mechanism and kinetics of GABA loading. Proc. Natl. Acad. Sci. U S A 113, 10702–10707. 10.1073/pnas.1604527113 27601664 PMC5035854

[B9] Eriksen J. Chang R. McGregor M. Silm K. Suzuki T. Edwards R. H. (2016). Protons regulate vesicular glutamate transporters through an allosteric mechanism. Neuron 90, 768–780. 10.1016/j.neuron.2016.03.026 27133463 PMC4886649

[B10] Farsi Z. Jahn R. Woehler A. (2017). Proton electrochemical gradient: driving and regulating neurotransmitter uptake. Bioessays 39, 1–9. 10.1002/bies.201600240 28383767

[B11] Farsi Z. Preobraschenski J. van den Bogaart G. Riedel D. Jahn R. Woehler A. (2016). Single-vesicle imaging reveals different transport mechanisms between glutamatergic and GABAergic vesicles. Science 351, 981–984. 10.1126/science.aad8142 26912364

[B12] Granseth B. Odermatt B. Royle S. J. Lagnado L. (2006). Clathrin-mediated endocytosis is the dominant mechanism of vesicle retrieval at hippocampal synapses. Neuron 51, 773–786. 10.1016/j.neuron.2006.08.029 16982422

[B13] Hori T. Takahashi T. (2012). Kinetics of synaptic vesicle refilling with neurotransmitter glutamate. Neuron 76, 511–517. 10.1016/j.neuron.2012.08.013 23141063

[B14] Lois C. Hong E. J. Pease S. Brown E. J. Baltimore D. (2002). Germline transmission and tissue-specific expression of transgenes delivered by lentiviral vectors. Science 295, 868–872. 10.1126/science.1067081 11786607

[B15] Martineau M. Guzman R. E. Fahlke C. Klingauf J. (2017). VGLUT1 functions as a glutamate/proton exchanger with chloride channel activity in hippocampal glutamatergic synapses. Nat. Commun. 8:2279. 10.1038/s41467-017-02367-6 29273736 PMC5741633

[B16] Rost B. R. Schneider F. Grauel M. K. Wozny C. Bentz C. Blessing A. (2015). Optogenetic acidification of synaptic vesicles and lysosomes. Nat. Neurosci. 18, 1845–1852. 10.1038/nn.4161 26551543 PMC4869830

[B17] Thévenaz P. Ruttimann U. E. Unser M. (1998). A pyramid approach to subpixel registration based on intensity. IEEE Trans. Image Process. 7, 27–41. 10.1109/83.650848 18267377

[B18] Tzingounis A. V. Wadiche J. I. (2007). Glutamate transporters: confining runaway excitation by shaping synaptic transmission. Nat. Rev. Neurosci. 8, 935–947. 10.1038/nrn2274 17987031

[B19] Yamashita M. Kawaguchi S. Y. Hori T. Takahashi T. (2018). Vesicular GABA uptake can be rate limiting for recovery of IPSCs from synaptic depression. Cell Rep. 22, 3134–3141. 10.1016/j.celrep.2018.02.080 29562170

[B20] Zhou Q. Petersen C. C. Nicoll R. A. (2000). Effects of reduced vesicular filling on synaptic transmission in rat hippocampal neurones. J. Physiol. 525, 195–206. 10.1111/j.1469-7793.2000.t01-1-00195.x 10811737 PMC2269926

[B21] Zimmermann J. Herman M. A. Rosenmund C. (2015). Co-release of glutamate and GABA from single vesicles in GABAergic neurons exogenously expressing VGLUT3. Front. Synaptic Neurosci. 7:16. 10.3389/fnsyn.2015.00016 26441632 PMC4585203

